# Postural Stability Margins as a Function of Support Surface Slopes

**DOI:** 10.1371/journal.pone.0164913

**Published:** 2016-10-20

**Authors:** Aviroop Dutt-Mazumder, Seymon M. Slobounov, John Henry Challis, Karl Maxim Newell

**Affiliations:** 1 Department of Kinesiology, The Pennsylvania State University, State College, PA, United States of America; 2 Department of Kinesiology, University of Georgia, Athens, GA, United States of America; University of Memphis, UNITED STATES

## Abstract

This investigation examined the effects of slope of the surface of support (35°, 30°, 20°, 10° Facing(Toe) Down, 0° Flat and 10°, 20°, 25° Facing (Toe) Up) and postural orientation on the margins of postural stability in quiet standing of young adults. The findings showed that the center of pressure—CoP (displacement, area and length) had least motion at the baseline (0° Flat) platform condition that progressively increased as a function of platform angle in both facing up and down directions. The virtual time to collision (VTC) dynamics revealed that the spatio-temporal margins to the functional stability boundary were progressively smaller and the VTC time series also more regular (SampEn–Sample Entropy) as slope angle increased. Surface slope induces a restricted stability region with lower dimension VTC dynamics that is more constrained when postural orientation is facing down the slope. These findings provide further evidence that VTC acts as a control variable in standing posture that is influenced by the emergent dynamics of the individual-environment-task interaction.

## Introduction

Upright standing posture is maintained by muscles that support the configuration of the whole body against gravity. This helps to stabilize the individual body elements to ensure that balance is preserved through the vertical projection of the center of gravity being within the base of support [1,2]. Upright postural balance has been largely assessed by variables of center of pressure (CoP) motion such as amplitude, velocity, acceleration, root-mean-square, scaling exponent and coefficient of variation [3–6]. However, more contemporary studies have illustrated the shortcomings of restricting analyses to CoP traces only [7]. The dynamics of the CoP have a dimension higher than that of a limit cycle oscillator [5,8], and reflect *1/f* properties [9] that are modulated with factors such as features of the individual, availability of feedback (environment), and the nature of the task base of support [10,11].

It has been postulated that the virtual time-to-collision (VTC) with the functional stability boundary could potentially be a low dimensional postural control variable [12]. VTC is defined as the time it would take, at any instant, for the CoP trajectory to reach the functional stability boundary. The computation of the VTC takes the current kinematics of the CoP and then predicts its motion given those kinematics until the CoP intercepts the functional stability boundary. Slobounov et al. (1997) derived the VTC in a two-dimensional plane (that can be extended to 3 D) to estimate postural stability, which reflects that the control variable for posture is defined by the organism-task-environment interaction [11, 13]. The coefficient of variation of VTC is typically lower than that of CoP position. It has been proposed that the VTC of the CoP with the functional stability boundary may yield important properties about the dynamic stability in upright postural balance [13]. The model illustrated the stability limits for center of mass (CoM) dynamics with respect to joint torques and state boundaries, and showed experimental support that torque safety margins were highly correlated with CoP safety margins, thereby supporting the use of CoP safety margins as a control variable for staying away from the functional stability boundary.

Standing on sloped surfaces represents a common postural challenge in human activity and CoP measures have been applied to investigate upright postural control to address the risk of slipping incidents owing to frictional demands [14]. Slopes have been employed to understand the after effects on leaning forward [15], the effects of slope on trunk kinematics during lifting weights [16], control of the fusimotor system [17] and perception-action coupling in infant posture [18]. Postural sway has been found to decrease in response to increased roof inclination [19], decrease in seamen using the horizon as a referent [20]. Furthermore, the effect of task specificity together with trade-offs between biomechanical and task constraint models of postural control have been shown in quiet standing [21].

The purpose of this study was to investigate the dynamic postural stability across sloped support base of platform angles, ranging from 35° Toe Down to 25° Toe Up, as a function of postural orientation. Given the above findings on postural control it was anticipated that the slope of the surface of support in relation to the orientation of the standing posture (toe up or down) would change the VTC dynamics in general and more specifically differentially relate to particular postural orientation segments of the functional stability boundary (e.g., fore, aft, side). Such a finding would provide further evidence that VTC acts as a control variable in standing posture that is influenced by the emergent dynamics of the individual-environment-task interaction.

Three hypotheses were examined. Firstly, we investigated the hypothesis that the stability index, which is a ratio of the actual CoP area (95% of the ellipsoid enclosed in CoP trace) to the functional stability boundary area would be closer to 0 for the 0° Flat platform condition. Secondly, based on previous studies [22,23], we examined the hypothesis that VTC would be lower in the extreme sloped platform conditions i.e. 35° Toe Down and 25° Toe Up when compared to 0° Flat platform condition. Thirdly, that the effect of slope angle on VTC dynamics changes as a function of the orientation of the posture to the slope (toe up or down) and hence the spatial region of the postural boundary. Collectively, the study investigated the postural stability margins across varying slope surfaces ranging from a flat support base to extreme angles of support base and how VTC properties contrast to standard CoP distributional metrics in the regulation of upright standing posture.

## Methods

### Participants

Seventeen healthy male participants provided written consent for their participation and were recruited, according to an experimental protocol approved by The Pennsylvania State University Institutional Review Board. Their heights ranged from 163 to 182 cm (mean = 174.5 cm), ages ranged from 23 to 37 years old, and the masses ranged from 55 to 89 kg (mean = 73 kg). All participants were healthy and self-reported no apparent neurological disorders and musculoskeletal injuries that could influence postural control.

### Instrumentation

An AMTI force platform (OPT400600-1000, Watertown, MA) was used to derive the displacement of the CoP. Pre-fabricated wooden platform wedges of different angles (25°, 20°, 10° Toe up (Up), 0° Flat, 10°, 20°, 30°, 35° Toe down (Down) were mounted on the force platform to provide slope surfaces. The slope angles were based on pilot tests of the biomechanical limits of ankle angle range of motion for the population group. The base of support on platform wedges was covered with commercial sand paper of grain size 100 to standardize the coefficient of friction across all platform angle conditions. The data were sampled at 100 Hz and were digitally low-pass filtered with a second order Butterworth filter with a cut-off frequency of 5 Hz.

### Tasks and procedures

The participants were instructed to maintain their upright postural balance when they stood on the force platform with bare feet, eyes open. They focused on a visual target placed 2 m away from the platform at eye level so as to remain as still as possible for the trial without further adjustments to the foot placement and maintaining their foot planted on the wedge for 40 s. The participant was familiarized with the protocol by a demonstration by an investigator followed by the participant practicing the task until they felt confident ~ <20 s. Thereafter, the platform angles were assigned randomly, with the baseline (0° Flat) as the first trial. Each platform condition had 2 trials, where the mean of the two trials were analyzed. There was a 1 min of recovery between successive trials and 4 min between each platform condition.

For each platform condition there was a trial to evaluate the functional stability boundary, where the participant was asked to lean as far as they could in 8 equally spaced directions within an ellipse surrounding the center of the base of support while maintaining the same foot placement throughout the entire trial [22].

### Data Analysis

CoP, which reflects the location of the ground reaction force at the surface of support, was analyzed along both *x* (AP) and *y* (ML) directions, with midpoint of the force plate as the origin [24]. There is a high correlation between several of the force platform CoP variables [3,10], and CoP area (95% confidence of ellipse area) which was applied to describe the degree of CoP motion exhibited by each participant. VTC was computed as in Slobounov et al. [22], which provides a direct measure of the relation of the CoP kinematics to the functional stability boundary.

The stability index, the ratio of the actual CoP area to the functional stability boundary area, was computed across the platform conditions. Sample Entropy (SampEn) was computed on the VTC time series to determine the irregularity of the VTC as a function of platform condition [22,23,24,25,26]. Input parameters for the SampEn calculation were: (1) a pattern length of 2 data points of VTC, and (2) a tolerance window normalized to 0.2 times the standard deviation of individual time series.

Statistical analysis of the data included descriptive statistics of the dependent variables and analysis of variance (ANOVA) of the variables across platform conditions. Significance was assumed when there was less than 5% chance for Type I error. When appropriate, Tukey’s post hoc analysis was performed to adjust the alpha value.

## Results

### Area of CoP

**Fig 1:**
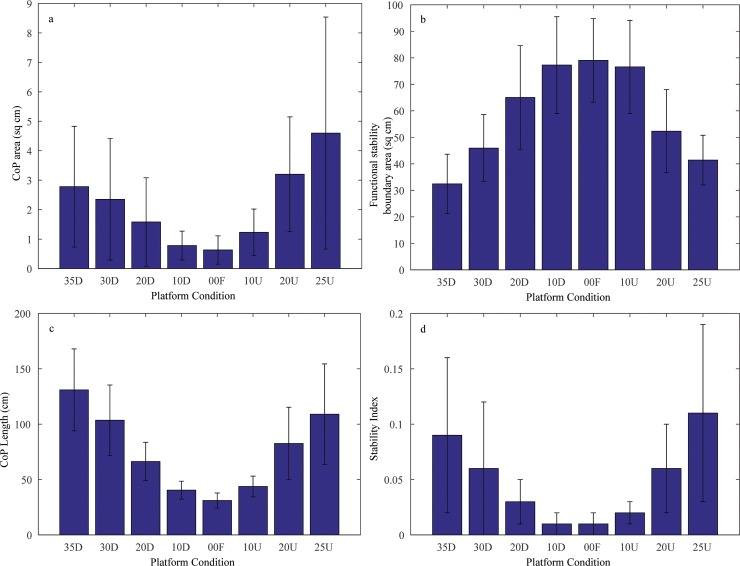
Group mean (*n =* 17) values with between-S standard deviation of: (a) CoP area, (b) Functional stability boundary area, (c) CoP length and (d) Stability index.

The area of CoP during quiet standing was smallest in the 0° Flat condition and CoP area was larger at 35° Down and largest at 25° Up condition (Fig 1A). In general, there was greater area to CoP motion in the toe Up condition (with more between-S variability) than the toe Down condition indicating asymmetry of control with postural orientation and slope. The effect of platform conditions on CoP area was significant, *F*(7,128) = 9.40 (*p*<0.05). Post-hoc Tukey analysis showed all pair-wise conditions were significant except for the following pairs 10° Down-10° Up, 35° Down-30° Down, 0° Flat-10° Down, 0° Flat-10° Up and 10° Up-20° Down.

### Functional Stability Boundary Area

The mean area of the functional CoP stability region as a function of slope condition is shown in Fig 1B. The effect of platform conditions on the functional stability area was significant, *F*(7,128) = 23.72 (*p*<0.05). Post-hoc Tukey analysis showed all pair-wise conditions were significant except for the following pairs: 10° toe Down-10° toe Up, 0° Flat-10° toe Up, 35° toe Down-20° toe Up, 35° toe Down-25° toe Up and 0° Flat-10° toe Down. The stability area was reduced at 25° Up and even more restricted at 35° Down condition.

### Length of CoP

For platform conditions 35° toe Down, 30° toe Down and 25° toe Up, the CoP length was larger than 1 m, whereas for platform condition 10° toe Down, 10° toe Up and 0° Flat, the length of CoP less than 0.5 m (see Fig 1C), thereby showing asymmetric change of CoP length as a function of platform condition. The effect of platform conditions on CoP length was significant, *F*(7,128) = 30.13 (*p*<0.05). Post-hoc Tukey analysis showed all pair-wise conditions were significant except for the following pairs: 10° toe Down-10° toe Up, 0° Flat-10° toe Up, 30° toe Down-25° toe Up and 0° Flat-10° toe Down.

### Stability Index

The histogram in Fig 1D shows that lower slope platform angles have a stability index closer to 0. The extreme slope platform angles have values between 0.1–0.15 with large variability across subjects. Platform angle had a significant effect on the stability index, *F*(7,128) = 11.60 (*p*<0.05). Post-hoc Tukey analysis showed all pair-wise conditions were significant except for the following pairs: 10° toe Down-10° toe Up, 0° Flat-10° toe Up, 20° toe Down-10° toe Up, 30° toe Down-20° toe Up and 0° Flat-10° toe Down.

### Virtual Time to Collision (VTC)

**Fig 2:**
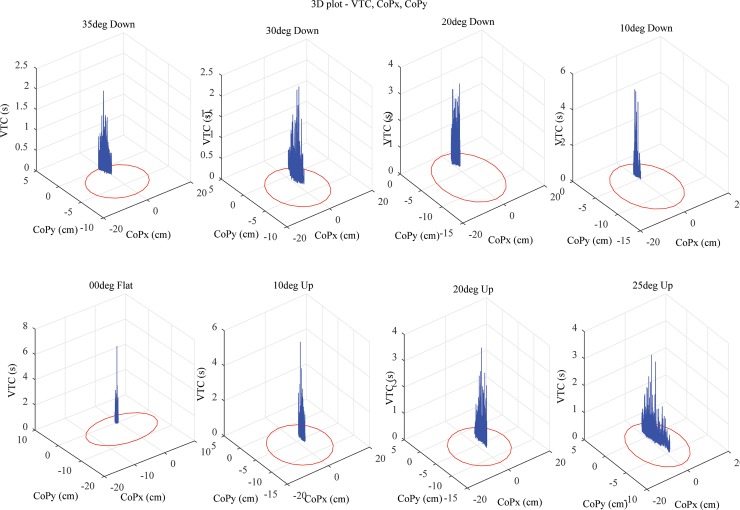
Representative 3D plot of VTC in CoPx and CoPy across all sloped platform conditions. The heel position is towards CoP*y* and the toes are oriented towards CoP*x*.

The functional stability boundary area, its orientation and distribution on the AP and ML axes is shown for a representative subject. The distribution is largest for 0° Flat platform condition, supplemented by reduced CoP distribution at the other slope platform conditions (see Fig 2). The representative plots are commensurable with the histogram plot of Fig 1A that shows the group mean (*n* = 17) of CoP area is higher with larger variability in the 25° toe Up platform condition than the 35° toe Down platform condition.

**Fig 3:**
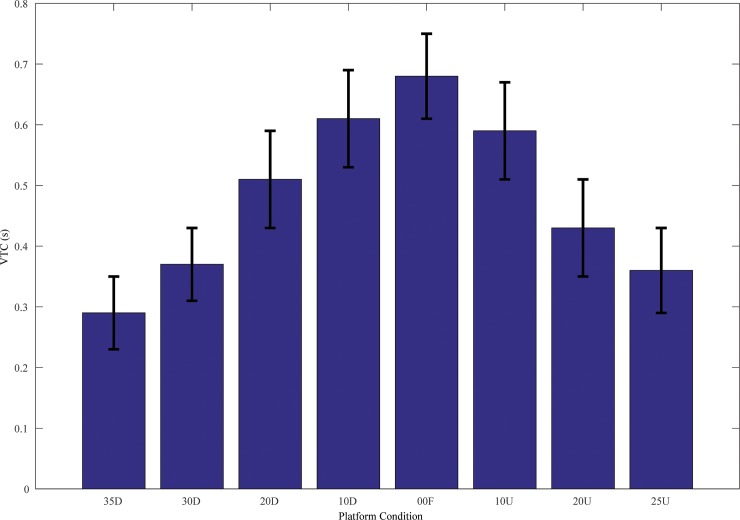
Representative 3D plot of VTC in CoPx and CoPy across all sloped platform conditions. The heel position is towards CoP*y* and the toes are oriented towards CoP*x*.

The mean VTC values are close to 0.7 s for 0° Flat platform condition and between 0.3 s to 0.4 s for the extreme slope platform conditions (35° toe Down and 25° toe Up) (see Fig 3). The nature of this change suggests that 0° Flat has highest VTC values and tapers down at the extreme platform slope conditions. The main effect of platform on VTC was significant, *F*(7,128) = 64.59 (*p*<0.05). Post-hoc Tukey analysis revealed that all pair-wise conditions were significant except for the following pairs 10° toe Down-10° toe Up, 35° toe Down-25° toe Up, 0° Flat-10° toe Up and 20° toe Up-20° toe Down.

### Virtual Time to Collision Irregularity (SampEn)

**Fig 4:**
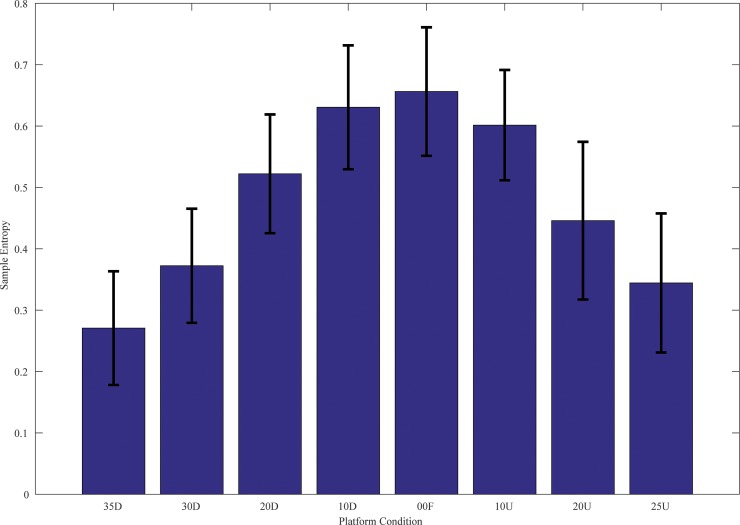
Group mean (n = 17) for SampEn with between-S standard deviation across all sloped platform conditions.

The mean SampEn across all the sloped platform conditions is shown in Fig 4. SampEn was higher for flat (0.66) and lower platform slopes and decreased to less than 0.35 for extreme platform angles. SampEn was significantly influenced by platform conditions, *F*(7,128) = 34.01 (*p*<0.05). Post-hoc Tukey analysis showed all pair-wise conditions were significant except for the following pairs: 10° toe Down-10° toe Up, 35° toe Down-25° toe Up, 0° Flat-10° toe Up, 0° Flat-10° toe Down and 20° toe Up-20° toe Down.

**Fig 5:**
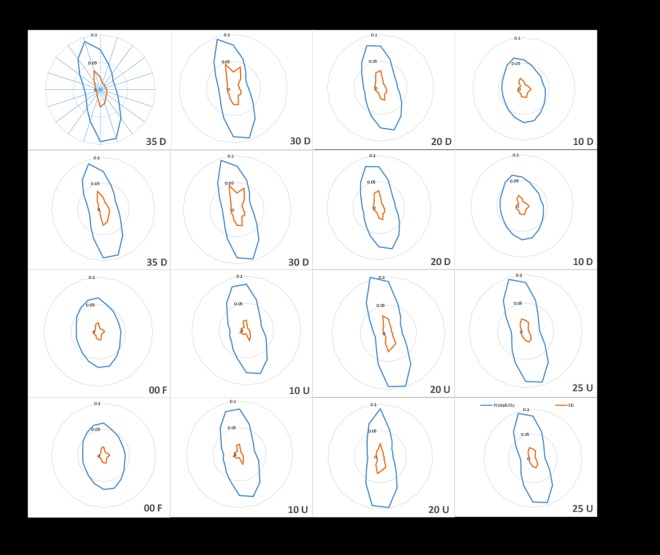
Group mean (n = 17) with between-S standard deviation for polar distribution of VTC mean magnitude and probability of a virtual contact across 20 boundary segments as a function of sloped platform conditions using CoP data. 1^st^ and 3^rd^ Row – VTC mean (s), 2^nd^ and 4^th^ Row – Probability of VTC.

The mean VTC values and the mean probability of virtual contacts for each of the 20 boundary segments is shown as CoP radar distribution with its corresponding standard deviation in Fig 5. There is a distinct qualitative change in the shape of the distributions across sloped platform angles. The radar distribution suggests that the CoP data have a wider spread at the lower sloped angles when compared with the elevated sloped platform angles. In addition to that, it can be noted that the CoP distribution is predominantly along AP direction and oriented towards the left side.

## Discussion

This study investigated postural regulation in young adults as a function of the postural orientation to the sloped angles of the surface of support. Upright postural control on sloped surfaces is a common postural challenge in daily human activity that is an emergent product of a complex dynamical system involving a large number of degrees of freedom [3,7,14,20]. The findings showed an interaction between slope and postural orientation (toe up or toe down) on the temporal and spatial margins of postural stability. In general, surface slope induced a restricted stability region with lower dimension VTC dynamics that was further constrained when participants were facing down the slope (toe down).

The CoP area was smallest in the 0° Flat, 10° toe Down and 10° toe Up platform conditions when compared with the higher sloped platform conditions. This is consistent with the finding that the mean area of CoP is significantly reduced when surface compliance is altered, i.e. standing on foam surface with no vision when compared to standing on firm ground with vision (10). Here the CoP length was largely restricted to less than .5 m for a group mean (*n* = 17) at 0° Flat, 10° toe Down and 10° toe Up platform conditions. In comparison the higher platform angles, i.e. 35° toe Down and 25° toe Up platform conditions had a CoP length twice than that of lower angle and flat platform conditions. In general, the distribution of CoP motion increases when a participant stands upright on an unaccustomed support base (e.g. foam surface or angled surface) in contrast to standing upright on a flat base of support.

To investigate the fluctuations of the stability index and VTC in sloped base of support the functional stability boundary was recorded across all platform angles. The functional stability boundary was restricted in overall area and particularly in the lateral direction with increments of slope of the support surface. There was also a small but general tilt to the left of the VTC orientation as a function of slope angle. There is evidence that the majority of the population have a right foot preference [27] and an associated right foot bias during standing tasks [28] which when standing on a slope will shift the center of pressure to the left side of a subject.

The functional stability boundary is a more appropriate procedure than the geometric boundary to calculate the stability index [29] as it incorporates the *actual* postural sway boundary on different platform angles of the individual and provides the *effective* boundary of stability for the posture. Here, the stability index being a dimensionless ratio number, designed to represent the postural stability on the sloped surface, which adjusts the values measured on different sloped angles to a notionally common scale that eliminates the effects of any anomaly in the VTC time series. The stability index was essentially conserved for flat and lower platform angles.

The results are consistent with previous findings on the postural stability index in studies of aging [23], support surface compliance [10] and vision/no vision with aging [30]. The CoP area and functional stability boundary area provide evidence on the sensitivity of spatial/temporal margins of postural stability to the sloped base of support and provide a boundary relevant measure for postural stability. This relative measure is revealing because the amount and location of CoP motion in the functional stability boundary does not directly relate to the temporal immediacy of making contact with the functional stability boundary [31].

The time series of VTC revealed that there was more time available on average to reach the functional stability boundary for flat and lower platform angle conditions. The sloped platform surfaces resulted in adaptation of postural sway dynamics to maintain upright postural stability thereby resulting in lower values of VTC and reduced stability. Thus, lower values of VTC can increase the probability of falling as a result of potentially a more immediate collision of the CoP with the functional boundary and the loss of stability [22]. These findings further support the hypothesis that postural control is regulated in relation to VTC with the functional stability boundary rather than minimization of the amount of postural sway around a CoP fixed point [12,19,27,28,29].

The complexity of spatio-temporal variable was highest in the flat condition even though CoP was restricted here to a smaller area and reduced length. The SampEn values were comparatively lower at the sloped platform conditions, thereby indicating that the predictability of VTC time series is more regular in such platform conditions. This supports the view that the task of quiet standing on a flat platform has a higher dimension than the sloped platform conditions that induce a lower dimension postural regularity for a spatio-temporal variable such as VTC, even when a spatial variable such as amount of motion of CoP is largely higher in such platform conditions [22].

There was an asymmetry in terms of the effect of toe up or toe down on the slope on the motion of CoP and its related variables that was statistically significant across the platform conditions. The stability index revealed that 25° toe Up platform condition was highly unstable with a larger CoP area. On the other hand, 35° toe Down condition had a more regular VTC time series with lower functional stability boundary and longer CoP length. Thus, the two extreme platform conditions had asymmetric postural sway properties consistent with the differential anatomical range of joint motion potential of fore and aft orientation [19]. The outcome is that spatial and temporal margins of postural stability are reduced with surface slope that is further magnified with a postural orientation that is toe down on the surface slope.

In summary, the study found that the stability index for a flat platform support condition and lower sloped platform conditions results in restricted CoP distribution complemented by higher safety margin values of VTC with more irregularity thereby indicating a higher dimension mechanism to control postural sway while maintaining upright equilibrium. On the other hand, the larger distribution of CoP traces along with lower values of VTC and SampEn suggest that a lower dimension mechanism is adopted to maintain upright postural equilibrium at sloped bases of support. The slope of the surface of support interacts with orientation in the organization and resultant sway of postural control.

These findings provide further evidence that VTC acts as a control variable in standing posture that is influenced by the emergent dynamics of the individual-environment-task interaction [10, 13, 22, 23, 32, 33]. The postural orientation as a function of the slope of the base of support significantly channels the emergent VTC dynamics independent of the standard metrics of the amount of motion of the CoP. The slope of the base of support induces postural dynamics that channel asymmetrically with postural orientation (toe up, toe down) the motion of the postural system toward the functional stability boundary.

## References

[1] King DL, Zatsiorsky VM. Extracting gravity line displacement from stabilographic recordings. Gait Posture [Internet]. 1997 8 [cited 2015 Jun 30];6(1):27–38.

[2] Rothwell J. Posture In: Control of Human Voluntary Movement. Netherlands: Springer; 1994 p. 252–92.

[3] Goldie PA, Bach TM, Evans OM. Force platform measures for evaluating postural control: reliability and validity. Arch Phys Med Rehabil [Internet]. 1989 7 1 [cited 2015 Aug 27];70(7):510–7. 2742465

[4] Murray MP, Seireg AA, Sepic SB. Normal postural stability and steadiness: quantitative assessment. J Bone Joint Surg Am [Internet]. The American Orthopedic Association; 1975 6 1 [cited 2015 Sep 25];57(4):510–6. 1141262

[5] Collins JJ, De Luca CJ. Open-loop and closed-loop control of posture: A random-walk analysis of center-of-pressure trajectories. Exp Brain Res [Internet]. 1993 8 [cited 2015 Apr 22];95(2):308–18. 8224055 10.1007/BF00229788

[6] Duarte M, Sternad D. Complexity of human postural control in young and older adults during prolonged standing. Exp brain Res [Internet]. 2008 11 [cited 2015 Sep 25];191(3):265–76. 10.1007/s00221-008-1521-7 18696056

[7] Ihlen EAF, Skjæret N, Vereijken B. The influence of center-of-mass movements on the variation in the structure of human postural sway. J Biomech [Internet]. 2013 2 1 [cited 2015 Sep 25];46(3):484–90. 10.1016/j.jbiomech.2012.10.016 23149080

[8] Myklebust, J. B., & Myklebust BM. Fractals in kinesiology. In: Society for Neuroscience Meeting. 1989. p. (Abstract no. 243.2).

[9] Duarte M, Zatsiorsky VM. On the fractal properties of natural human standing. Neurosci Lett [Internet]. 2000 4 [cited 2015 Sep 25];283(3):173–6. 10754215 10.1016/s0304-3940(00)00960-5

[10] Haibach PS, Slobounov SM, Slobounova ES, Newell KM. Virtual time-to-contact of postural stability boundaries as a function of support surface compliance. Exp brain Res [Internet]. 2007 3 [cited 2015 Sep 25];177(4):471–82. 10.1007/s00221-006-0703-4 17031683

[11] Newell KM. Constraints on the development of coordination In: Whiting MG& WHT., editor. Motor development in children: Aspects of coordination and control. Amsterdam: Martinus Nijhoff Publishers; 1986 p. 341–61.

[12] Carello C, Turvey MT, Kugler PN. The informational support for upright stance. Behav Brain Sci [Internet]. Cambridge University Press; 1985 2 4 [cited 2015 Sep 25];8(1):151.

[13] Patton JL, Pai Y-C, Lee WA. Evaluation of a model that determines the stability limits of dynamic balance. Gait Posture [Internet]. 1999 3 [cited 2015 Aug 11];9(1):38–49. 10575069 10.1016/s0966-6362(98)00037-x

[14] Zhao Y, Upadhyaya SK, Kaminaka MS. Foot-ground forces on sloping ground when lifting. Ergonomics [Internet]. Taylor & Francis Group; 1987 12 31 [cited 2015 Sep 25];30(12):1671–87. 10.1080/00140138708966057 3443091

[15] Kluzik J, Horak FB, Peterka RJ. Differences in preferred reference frames for postural orientation shown by after-effects of stance on an inclined surface. Exp brain Res [Internet]. 2005 5 [cited 2015 Sep 25];162(4):474–89. 10.1007/s00221-004-2124-6 15654594

[16] Shin G, Mirka G. The effects of a sloped ground surface on trunk kinematics and L5/S1 moment during lifting. Ergonomics [Internet]. Taylor & Francis Ltd; 2004 5 15 [cited 2015 Sep 25];47(6):646–59. 10.1080/00140130310001653066 15204292

[17] Aniss AM, Diener HC, Hore J, Gandevia SC, Burke D. Behavior of human muscle receptors when reliant on proprioceptive feedback during standing. J Neurophysiol [Internet]. 1990 8 1 [cited 2015 Sep 25];64(2):661–70. 2213138 10.1152/jn.1990.64.2.661

[18] Adolph KE, Eppler MA, Marin L, Weise IB, Wechsler Clearfield M. Exploration in the service of prospective control. Infant Behav Dev. 2000;23(3):441–60.

[19] Emerich, R., Bhattacharya, A., Succop, P. A., & Bagchee A. Effect of roof inclination on postural stability and perceived sense of fall. In: Annual American Industrial Hygiene Conference and Exposition. 1993. p. 104.

[20] Mayo AM, Wade MG, Stoffregen TA. Postural effects of the horizon on land and at sea. Psychol Sci [Internet]. SAGE Publications; 2011 1 [cited 2016 Sep 16];22(1):118–24. 10.1177/0956797610392927 21156861

[21] Riley M. A., Mitra S., Stoffregen T. A., & Turvey MT. Influences of body lean and vision on unperturbed postural sway. Motor Control. 1997;1(3):229–46.

[22] Slobounov SM, Slobounova ES, Newell KM. Virtual Time-to-Collision and Human Postural Control. J Mot Behav [Internet]. Taylor & Francis Group; 1997 9 2 [cited 2015 Sep 25];29(3):263–81. 10.1080/00222899709600841 12453785

[23] Slobounov SM, Moss SA, Slobounova ES, Newell KM. Aging and Time to Instability in Posture. Journals Gerontol Ser A Biol Sci Med Sci [Internet]. 1998 1 1 [cited 2015 Sep 25];53A(1):B71–80. 10.1093/gerona/53a.1.b71 9467425

[24] Winter DA. Biomechanics and motor control of human movement New Jersey: John Wiley & Sons, Ltd.; 2009.

[25] Donker SF, Roerdink M, Greven AJ, Beek PJ. Regularity of center-of-pressure trajectories depends on the amount of attention invested in postural control. Exp Brain Res [Internet]. 2007 3 31 [cited 2015 Oct 7];181(1):1–11. 10.1007/s00221-007-0905-4 17401553 PMC1914290

[26] Ramdani S, Seigle B, Lagarde J, Bouchara F, Bernard PL. On the use of sample entropy to analyze human postural sway data. Med Eng Phys [Internet]. 2009 10 [cited 2015 Oct 7];31(8):1023–31. 10.1016/j.medengphy.2009.06.004 19608447

[27] Chapman JP, Chapman LJ, Allen JJ. The measurement of foot preference. Neuropsychologia [Internet]. 1987 [cited 2016 Sep 16];25(3):579–84. 3683814 10.1016/0028-3932(87)90082-0

[28] Gutnik B, Leaver J, Standen C, Longley C. Inferred influence of human lateral profile on limb load asymmetry during a quiet standing balance test. Acta Med Okayama [Internet]. 2008 6 [cited 2016 Sep 16];62(3):175–84. 18596834 10.18926/AMO/30983

[29] Holbein MA, Redfern MS. Functional stability limits while holding loads in various positions. Int J Ind Ergon [Internet]. 1997 5 [cited 2015 Sep 22];19(5):387–95. 11540602 10.1016/s0169-8141(96)00023-6

[30] Slobounov SM, Haibach PS, Newell KM. Aging-related temporal constraints to stability and instability in postural control. Eur Rev Aging Phys Act [Internet]. 2006 8 10 [cited 2015 Sep 27];3(2):55–62.

[31] Kilby MC, Slobounov SM, Newell KM. Postural instability detection: aging and the complexity of spatial-temporal distributional patterns for virtually contacting the stability boundary in human stance. PLoS One [Internet]. Public Library of Science; 2014 1 8 [cited 2016 Mar 18];9(10):e108905 10.1371/journal.pone.0108905 25295589 PMC4189796

[32] Riccio G. Information in movement variability about the qualitative dynamics of posture and orientation In: Newell K, Corcos D, editors. Variability and motor control. Champaign, IL: Human Kinetics; 1993 p. 317–58.

[33] van Wegen EE, van Emmerik RE, Wagenaar RC, Ellis T. Stability boundaries and lateral postural control in parkinson’s disease. Motor Control [Internet]. 2001 7 1 [cited 2015 Sep 27];5(3):254–69. 11438764 10.1123/mcj.5.3.254

